# NLX-112, a Selective 5‑HT_1A_ Receptor Agonist, Upregulates GDNF and is Neuroprotective Against MPTP-Induced Nigrostriatal Degeneration in a Mouse Model of Parkinson’s Disease

**DOI:** 10.1021/acschemneuro.6c00251

**Published:** 2026-07-02

**Authors:** William H. Powell, Lucy E. Annett, Ronan Depoortere, Adrian Newman-Tancredi, Mahmoud M. Iravani

**Affiliations:** † Department of Medicine, Applied and Clinical Sciences, College Lane, University of Hertfordshire, Hatfield AL109AB, U.K.; ‡ Department of Psychology, Sport and Geography, School of Health, Medicine and Life Sciences, College Lane, University of Hertfordshire, Hatfield AL109AB, U.K.; § Neurolixis SAS, 2 Rue Georges Charpak, Castres 81100, France

**Keywords:** Neuroprotection, NLX-112, 5-HT_1A_, MPTP, GDNF, open field, thigmotaxis, locomotor activity, mouse model of Parkinson’s disease, microglia, astrocytes

## Abstract

NLX-112 is a potent and selective 5-HT_1A_ agonist that has successfully completed a Phase 2A clinical trial for the treatment of L-DOPA-induced dyskinesia in Parkinson’s disease (PD) patients. Here, we investigated the neuroprotective activity of NLX-112 in a mouse 1-methyl-4-phenyl-1,2,3,6-tetrahydropyridine (MPTP) model of PD. Four groups of male mice received subcutaneous administrations of either saline (1 mL/kg) daily for 15 days, MPTP (21.4 mg/kg for 5 days, preceded and followed by 5 days of saline, NLX-112 (1 mg/kg/day for 15 days), or combined MPTP + NLX-112. Two weeks following the cessation of treatments, mice treated with NLX-112 alone or NLX-112/MPTP showed increased locomotor activity and reduced anxiety-like behavior (as assessed by a 40% reduction of thigmotaxis) in an open-field test, consistent with sustained effects of 5-HT_1A_ receptor activation. MPTP-treated mice showed a 40% reduction of dopaminergic (i.e., tyrosine hydroxylase immunoreactive; TH-ir) neurons in the substantia nigra compacta (SNc) and a 55% reduction of nerve terminals in the striatum. NLX-112 treatment completely prevented TH-ir neuron loss and reduced MPTP-induced dopamine nerve terminal loss to 30%. MPTP also markedly increased the levels of GFAP-ir astrocytes and Iba1-ir microglia in the SN, as well as coexpression of glial-derived neurotrophic factor (GDNF) in the GFAP-ir astrocytes in both the SNc and the striatum. NLX-112 markedly reversed this MPTP-induced microglial overactivation in the SNc, and upregulated GFAP/GDNF colocalization in both the striatum and the SNc. Overall, the present study demonstrates a robust neuroprotective effect of NLX-112 in a mouse model of PD by preventing microgliosis, upregulating GDNF, and favoring sustained pro-locomotor activity.

## Graphical abstract

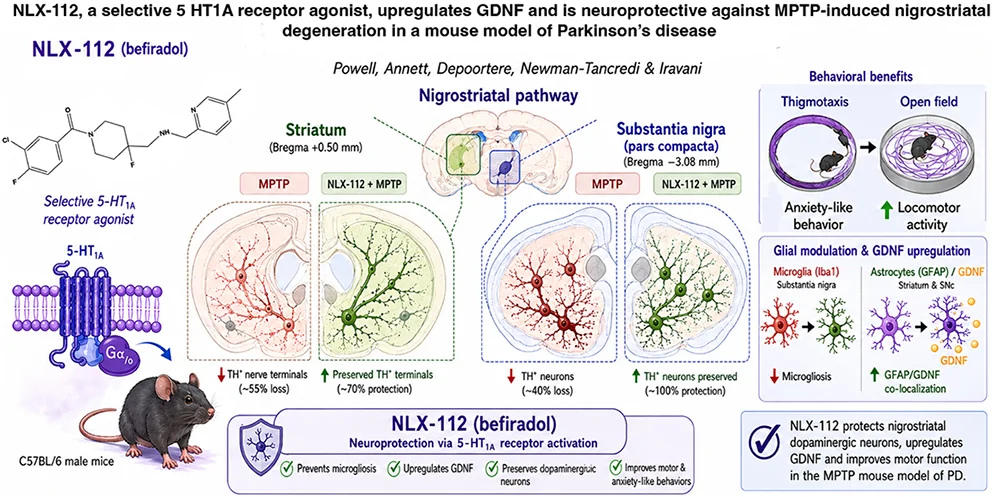

## Introduction

NLX-112 is a first-in-class, highly selective, and potent 5-HT_1A_ receptor agonist that effectively counteracts dyskinesia and abnormal involuntary movements in levodopa-primed rodent and primate models of Parkinson’s disease (PD).^1–4^ Moreover, a recent double-blind, placebo-controlled Phase 2A clinical trial demonstrated robust antidyskinetic and antiparkinsonian efficacy of NLX-112 in PD patients.^5^ Beyond its efficacy for the treatment of these motor symptoms, NLX-112 may also constitute a promising candidate for disease modification through neuroprotection. Indeed, progressive degeneration of dopaminergic neurons in the substantia nigra (SN) remains the pathological hallmark of PD, and no approved therapy halts or reverses this process. The identification of pharmacological agents capable of preserving dopaminergic neurons is, therefore, a key research priority.

A potential strategy for neuroprotection in PD involves targeting the serotonergic system because this is known to modulate neuronal excitability, synaptic activity, and glial responses within the basal ganglia.^6^ Substantial evidence supports a neuroprotective role for 5-HT_1A_ receptor activation. The prototypical 5-HT_1A_ receptor agonist, 8-OH-DPAT, prevents oxidative-stress-induced neuronal death by reducing Na^+^ and Ca^2+^ influx, thereby attenuating glutamate release and excitotoxicity.^7,8^ It also inhibits NMDA receptor-mediated caspase-3 activation and DNA fragmentation.^9^ Similarly, two other 5-HT_1A_ receptor agonists, repinotan (BAYx3702) and BAY639044, protect neurons in vitro and in MPTP-lesioned animals through activation of MAPK- and NGF-dependent pathways that suppress apoptosis.^10–12^ Partial agonists, such as buspirone and ipsapirone, also engage PI3K and MAPK signaling cascades to promote neuronal survival.^13^

The astroglial contribution to 5-HT_1A_ receptor-mediated neuroprotection is increasingly recognized. Activation of astroglial 5-HT_1A_ receptors modulates inflammatory signaling and enhances the release of neuroprotective factors. For example, 8-OH-DPAT reduces astrocyte density and IL-1β levels in toxin-exposed brain regions.^14^ Furthermore, rotigotine, a dopamine agonist with a dual mechanism that includes activity at 5-HT_1A_ receptors, upregulates metallothionein expression in striatal astrocytes, thereby protecting dopaminergic neurons in a WAY100635-dependent manner from 6-OHDA toxicity.^15,16^ These findings support a mechanism in which astroglial–neuronal crosstalk, mediated through 5-HT_1A_ receptor signaling, mitigates oxidative stress, inflammation, and apoptosis within dopaminergic circuits.

NLX-112 (a.k.a. befiradolor F13640) is characterized by nanomolar affinity and biased agonism at the 5-HT_1A_ receptor, preferentially coupling to Gαo protein signaling.^17–19^ Its intrinsic efficacy equals that of serotonin and exceeds that of earlier agonists such as 8-OH-DPAT and buspirone.^18–20^ NLX-112 exhibits exceptional receptor selectivity, with no detectable affinity for dopaminergic, adrenergic, GABAergic, or other serotonergic subtypes, and shows no interaction with monoamine oxidases.^17^ Together with its favorable CNS-penetrant pharmacokinetics, these properties position NLX-112 as a mechanistically rational candidate for neuroprotection in PD. The present study, therefore, investigates whether activation of 5-HT_1A_ receptor-dependent signaling by NLX-112 confers protection against dopaminergic neurodegeneration in the MPTP mouse model of PD, using behavioral, histological, and biochemical endpoints to define its neuroprotective efficacy and mechanism of action.

## Experimental Methods

### Drugs

NLX-112 (3-Chloro-4-fluorophenyl-[4-fluoro-4-([(5-methylpyridin-2-yl)­methylamino] methyl)­piperidin-1-yl]­methanone, fumarate salt) was provided by Neurolixis and prepared daily in a vehicle consisting of saline (0.9% NaCl) and 0.25% dimethyl sulfoxide (DMSO) at a working solution of 1 mg/8 mL. 1-Methyl-4-phenyl-1,2,3,6-tetrahydropyridine hydrochloride (MPTP) was purchased from Sigma–Aldrich (UK) and diluted with saline on the day of the experiment. Doses are expressed as the weight of the free base.

### Animals

All experiments adhered to the ARRIVE guidelines and were conducted in compliance with the UK Animals (Scientific Procedures) Act 1986 and EU directive 2010/63/EU for animal experiments. The University of Hertfordshire Animal Welfare and Ethical Review Board (AWERB) approved all procedures and protocols, operating under the UK Home Office-approved Project License PD7255B4. Male mice (strain: C57blJ6-OlaHSD, RRID: IMSR_ENV:HSD-057), aged 8–10 weeks (19–21g) at arrival, were purchased from Envigo Laboratories (now Inotiv, UK). This strain of mice was chosen as it is more resilient to the effects of MPTP and exhibits less weight loss during MPTP treatment.^21^ The sample sizes were chosen based on previous behavioral investigations in mice^21,22^ in line with NC3R’s recommendations for justification of animal numbers. Mice were housed in groups of 3 to 4 in single-use mouse plastic disposable cages (SUMC, floor area 501 cm^2^; Tecniplast, UK). Access to food and water was ad-libitum, room temperature was maintained at 22 °C ± 2 °C, and the light cycle was automated at 12 h:12 h (light)/dark cycle). All experiments were carried out under an ambient light intensity of 100 lux between 09:00 h and 14:00 h.

### NLX-112 and MPTP Treatments

A total of forty-eight mice were used in this study. Animals were randomly allocated to one of four experimental groups (*n* = 12 per group): (A) vehicle-treated (NaCl + 0.25% DMSO, i.p.) for 15 consecutive days, with saline administered subcutaneously (s.c.) from day 6 to day 10; (B) vehicle-treated for 15 days, with MPTP administered s.c. from day 6 to day 10; (C) NLX-112-treated (i.p.) for 15 days, with saline administered s.c. from day 6 to day 10; and (D) NLX-112-treated for 15 days, with MPTP administered s.c. from day 6 to day 10 (Figure 1).

**Figure 1:**
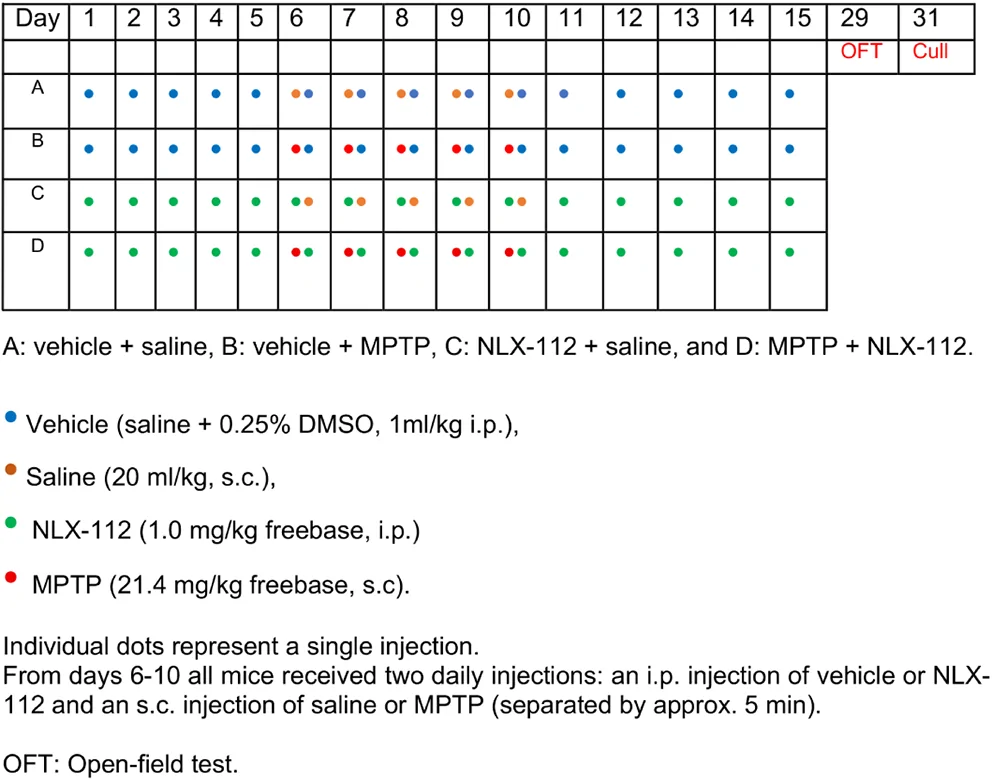
Dosing regimen for treatments.

MPTP administration is associated with acute tachycardia and rapid activation of adrenergic receptors in cardiac and vascular tissues, resulting in vasoconstriction, hypothermia, and an increased risk of cardiovascular failure and mortality.^23^ To minimize these adverse effects and refine animal welfare outcomes in accordance with the NC3Rs principles, MPTP was administered using an incremental, subchronic dosing regimen (single s.c. injection per day), allowing for desensitization of peripheral and vascular adrenergic receptors. This approach resulted in the 100% survival of MPTP-treated animals.

Due to the risk of cross-contamination, MPTP-treated animals were housed separately from non–MPTP-treated animals. As a result, full blinding of treatment allocation was not feasible. This limitation is explicitly acknowledged in accordance with ARRIVE 2.0 guidance. To mitigate potential assessment bias, animals were arbitrarily assigned to behavioral testing blocks, with each block containing one animal from each treatment group (A–D), ensuring balanced representation across testing sessions.

### Video Tracking and Open Field Test

The open-field test (OFT) was performed on day 29, i.e., 14 days after the end of the pharmacological treatment period, before the mice were euthanized. All open-field behavioral assessments were carried out using a Basler infrared camera (Basler AG, Germany), infrared light boxes, and analyzed with the Noldus Ethovision XT (RRID:SCR_000441) tracking software (Tracksys, Nottingham, UK) according to previously published methods.^22^

Locomotor activity was determined by the extent of exploratory movements in a 40 × 40 cm arena. In a novel environment, mice display thigmotaxis, a tendency to remain close to the walls of an arena that is used as a measure of anxiety in this species.^21,22^ To determine the extent of thigmotaxis, a 30 × 30 cm central zone in the center of the arena was digitally created with the Ethovision-XT software, leaving a 5 cm wide channel at the perimeter of the arena. The following parameters were measured using the Ethovision-XT software: total distance traveled (cm); stop-start frequency, mean velocity (cm/s), frequency of acceleration in the center of the arena, number of entries into the center arena from the edge, duration of movement into the center, and time spent in the center. Mice were placed in the arena for a total of 5 min.

### Immunohistochemistry

Mice were culled on Day 31 by exposure to CO_2_ according to NIH guidelines (CO_2_ flow rate: 5–6 L/min) in a Vet-Tech medium red chamber (Vet–Tech, UK), transcardially perfused with ice-cold phosphate-buffered saline (PBS), decapitated, their brains fixed in formalin (10% buffered), and prepared accordingly for immunohistochemical analyses. Brains were sectioned coronally at 30 μm thickness and analyzed for avidin–biotin visible light IHC for detection of nigral tyrosine hydroxylase (TH)-immunoreactive (−ir) neurons and stereology. Immunofluorescence immunohistochemistry was carried out to detect TH-ir axon terminals in the striatum, astrocytes, and microglia using sheep tyrosine-hydroxylase (TH), at 1:500 (Thermo Fisher Scientific Cat# PA1–4679, RRID:AB_561880), chicken glial fibrillary associated protein (GFAP) at 1:1000 (Thermo Fisher Scientific Cat# PA1–10004, RRID:AB_1074620), and rabbit ionized calcium-binding adapter molecule 1 (Iba1) at 1:500 dilution (Abcam Cat# ab178847, RRID:AB_2832244), respectively. We also examined the possibility of the presence of glial-derived neurotrophic factor (GDNF), as we have observed previously to be colocalized with astrocytes^24,25^ using a rabbit polyclonal GDNF antibody used at 1:500 dilution (Thermo Fisher Scientific Cat# PA5–89957, RRID:AB_2805864). Free-floating sections were washed in PBS (1X), with triton X-100 (0.1%), nonspecific binding was inhibited with a nonanimal protein blocker (Vector), and incubated overnight in the primary antibody at 4 °C. The following day, sections were washed and incubated in the secondary antibody for 1 h at room temperature (20–22 °C). The relevant secondary antibodies were tagged with AlexaFluor 594 (donkey antisheep, Thermo Fisher Scientific Cat# A-11016, RRID:AB_2534083, (goat antirabbit, Thermo Fisher Scientific Cat# A-11012, RRID:AB_2534079) and AlexaFluor 488 (goat antichicken, Thermo Fisher Scientific Cat# A-11039, RRID:AB_2534096). For avidin-biotin reactions, 3,3-diaminobenzidine (DAB) was used as a chromogen (DAB kit from Vector). Tissue was mounted on slides, and images were taken on a Zeiss Axiophot light microscope and photographed with a Zeiss AxioCam SE.

Cell counting in the substantia nigra was performed by either counting every cell that was TH-ir at the levels of the third cranial nerve^26^ or the total number of TH-ir neurons were estimated by stereological quantification.^100^ From within the boundaries of the nigrostriatal bundle (∼Bregma −2.50) and the caudal portion of the SNc (∼Bregma −3.90), five TH-ir sections, 300 μm apart (every 10th section), were observed, and cells were digitally identified and counted using ImageJ. The following calculation was used to achieve the final estimate of TH-ir neurons.

$$N=\overset{5}{\underset{i=1}{\sum }}\left(A_{i}\times ssf\right)\times mcf$$

The estimated number of cells (N) has been derived, where Σ is the sum of sections*(i)* ranging from 1 to 5 (Σ^5^ *i)*. *ssf* (=10) is the sampling fraction, which is one section analyzed every 10 sections. *mcf* is the missing cell fraction of uncounted cells; cells lie on their side or are layered on top of each other through the Z-axis = 1.33.

### Optical Density Measurement

To measure the extent of staining, TH-ir staining in the striatum, striatal digital images taken at x2 magnification were converted into 8-bit grayscale images and compared against a grayscale stepladder, from which a calibration curve was obtained. To assess the extent of nerve terminal loss in the striatum, the integrated optical density of striatal sections (3–4 striatal sections per animal) was processed for fluorescence TH-ir. To determine the extent of striatal TH-ir following MPTP or vehicle, the extent of TH-immunostaining was assessed from areas within the striatum using the entire visual field at x20 magnification at 5 to 8 sites. Variations in staining intensity within the dorsal caudate putamen subregions^27^ were compared in different animal groups using ImageJ image analysis software.

### Data Analysis and Statistics

For the analysis of each parameter of the OFT, and for the analysis of quantitative immunohistochemical data, we used a mixed model ANOVA, which allows handling of missing values. When a statistically significant difference was observed in the ANOVA, the data were further analyzed using Tukey’s *post hoc* test. Differences between group means were considered statistically significant when *p* < 0.05. All data sets were first tested for normality of distribution using QQ-plots using the D’Agostino-Pearson omnibus (K2) test. Normality of distribution was considered passed when alpha = 0.05 or larger. Statistical analyses were performed using GraphPad Prism v10.4 (RRID:SCR_002798).

## Results

### Behavior in the Open-Field Test

The distance traveled by mice that had received both NLX-112 (1 mg/kg) and MPTP was more than double that traveled by vehicle/saline-treated mice [F­(4, 50) = 15.76, *P* < 0.0001, Figure 2A)]. Similarly, the frequency of stop/starts [F­(4,46)=18, *P* < 0.0001, Figure 2B], velocity [F­(3, 36)=20.97, *P* < 0.0001, Figure 2C], and acceleration in the center of the arena [F­(4, 51)=11.59, *P* < 0.0001, Figure 2D] were significantly increased by this treatment. Entries into the center of the cage, which represent a reduction in anxiety-like behavior^22^ were increased significantly [F­(4, 51)=11.53, *P* < 0.0001, Figure 2E] so was the duration of movement in the center [F­(4, 40)=13.41, *P* < 0.0001, Figure 2F]. MPTP treatment significantly slowed the vehicle- and NLX-112-treated mice. However, in unlesioned and MPTP-lesioned animals, NLX-112-treatment significantly increased distance traveled and frequency of stop/starts (Figure 2A,B). Following MPTP, acceleration in the center of the arena, entries into the center of the arena, and duration of movement in the center of the arena were significantly increased by NLX-112 (Figure 2D–F).

**Figure 2:**
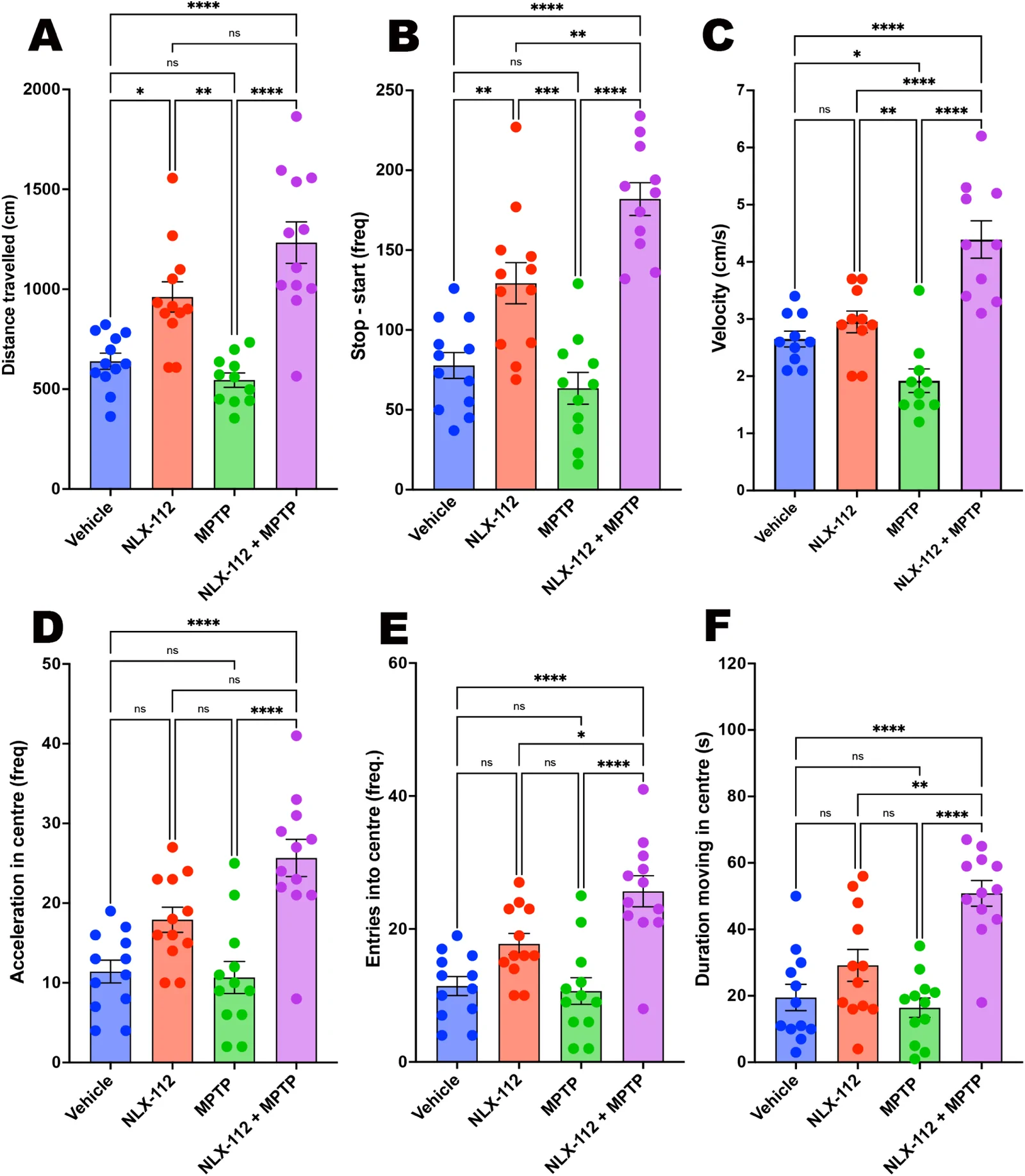
Behavior over 5 min in the open-field test, determined following 14 days of treatment cessation. A) Total distance traveled. B) Frequency of stop/starts, C) velocity, D) frequency of episodes of acceleration, E) frequency of entries into the center of the arena. F) Time spent in the center of the arena. *P < 0.05, **P < 0.001,*P* < 0.0005, ****P < 0.0001, Tukey’s post hoc test, following significant one-way ANOVA. *N* = 10–12 per group, bars are means; error bars are SEMs.

### TH-ir in SNc Dopaminergic Neurones and Projecting Fibres

MPTP treatment led to a reduction of TH-ir (i.e., dopaminergic) neurons in the caudal portion of the SN (SNc) (Figure 3A,B) and a loss of TH-ir nerve fibers in the striatum (Figure 3D). Nigral TH-ir neurons were first counted manually at the level of the third cranial nerve. There was an overall statistical difference in the number of TH-ir neurons at this level of the SNc following different treatments [F­(4, 33) = 2.932, *P* = 0.0352]. Treatment with NLX-112 alone had no effect on TH-ir cell numbers in the SNc or on TH-ir fiber density in the striatum.

**Figure 3:**
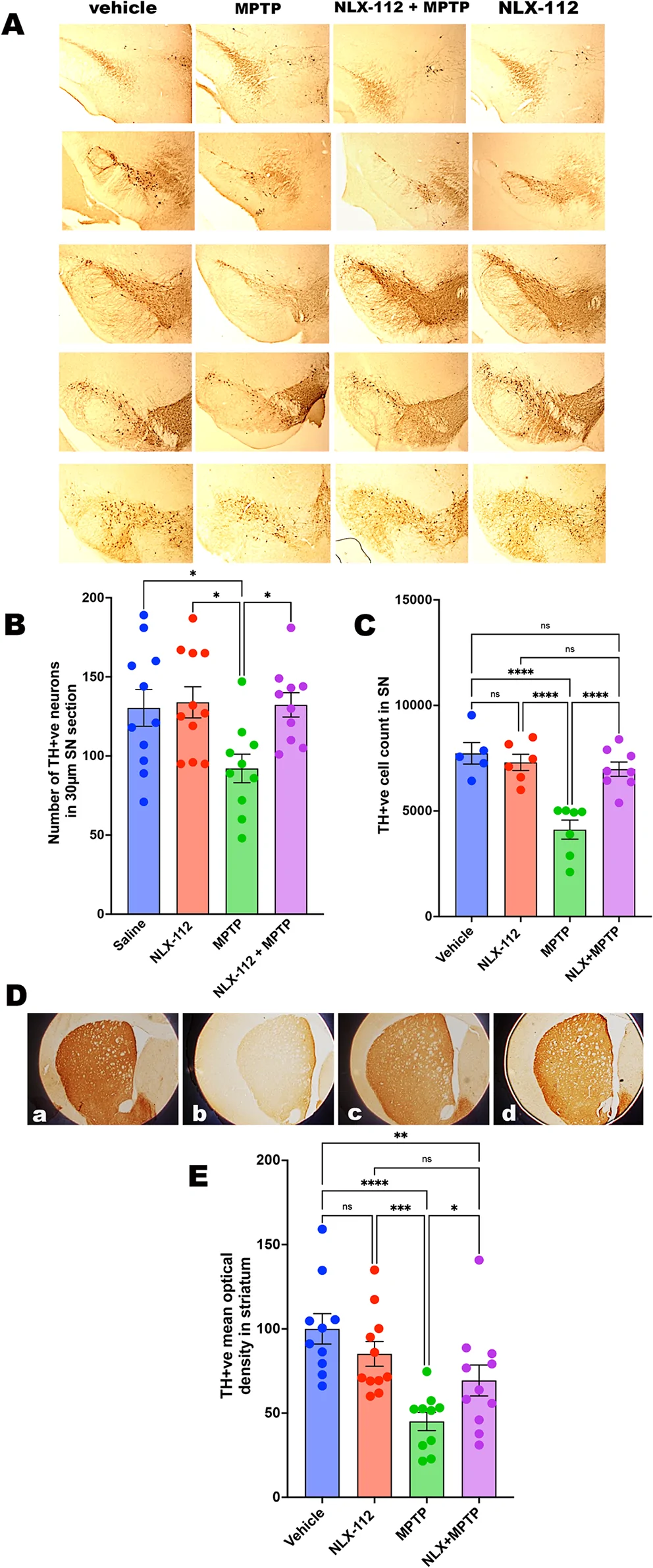
A) Representative images of TH-ir stained Substantia nigra pars compacta (SNc). Sections were taken from Bregma −2.50 at the nigrostriatal bundle through to Bregma −3.90 at the caudal portion of the SN. **B)** TH-ir neuronal counts at the level of the 3rd cranial nerve (*n* = 5–7). **C)** The total number of TH-ir cells stereologically counted in SN and **D)** representative striatal sections where a represents vehicle treatment, b MPTP, c combination of MPTP + NLX-112 and d is NLX-112 alone. The mean optical density of TH-ir fibers in the CPU is shown in panel **E**. *P < 0.05, **P < 0.001, ***P < 0.0001, Tukey’s post hoc-test following significant one-way ANOVA. *N* = 10–12 per group, bars are means; error bars are SEMs.

NLX-112 fully reversed the MPTP-induced loss of TH-ir neurons (Figure 3B,C). The total estimate of TH-ir neurons in the SNc following saline, NLX-112, and MPTP+NLX-112 is shown in Figure 3C. MPTP caused an approximately 40% loss of these neurons compared to the vehicle/saline treatment and saline/NLX-112 groups. Notably, NLX-112 treatment in the MPTP group completely protected TH-ir neurons in this brain region [F­(3, 22) = 15.67, *P* < 0.0001, Figure 3C]. Protective effects were also observed in the striatum, where MPTP caused a 55% loss in TH-ir fiber density, whereas NLX-112 attenuated this loss to 30%, compared to vehicle treatment [F­(3, 39) = 8.428, *P* = 0.0002, Figure 3D,E].

### Reactive Microgliosis and Iba-1 and TH Colocalization

Immunohistochemical analysis showed the presence of numerous Iba1-ir cells in the SNc (Figure 4A,C). In the NLX-112 alone group, the number and morphology of these cells did not change. Double immunofluorescence of Iba-1-ir and TH-ir cells showed that Iba-1-ir cells were much smaller and interleaved among TH-ir neurons. In the MPTP group, the loss of TH-ir neurons (see above) coincided with an extensive increase in the number of Iba-1-ir microglial cells and a change of morphology from fibrillar to a more condensed and occasionally amoeboid shape. The areas in the SNc that were devoid of TH-ir had more activated, condensed, and larger Iba-1-ir cells. NLX-112 completely reversed the drastic MPTP-induced increase in the number of activated Iba-1-ir cells [F­(3, 18) = 18.60, *P* < 0.0001, Figure 4B], and the morphology of Iba-1-ir cells became more fibrillar. Very few Iba-1-ir cells were observed in the striatum (data not shown).

**Figure 4:**
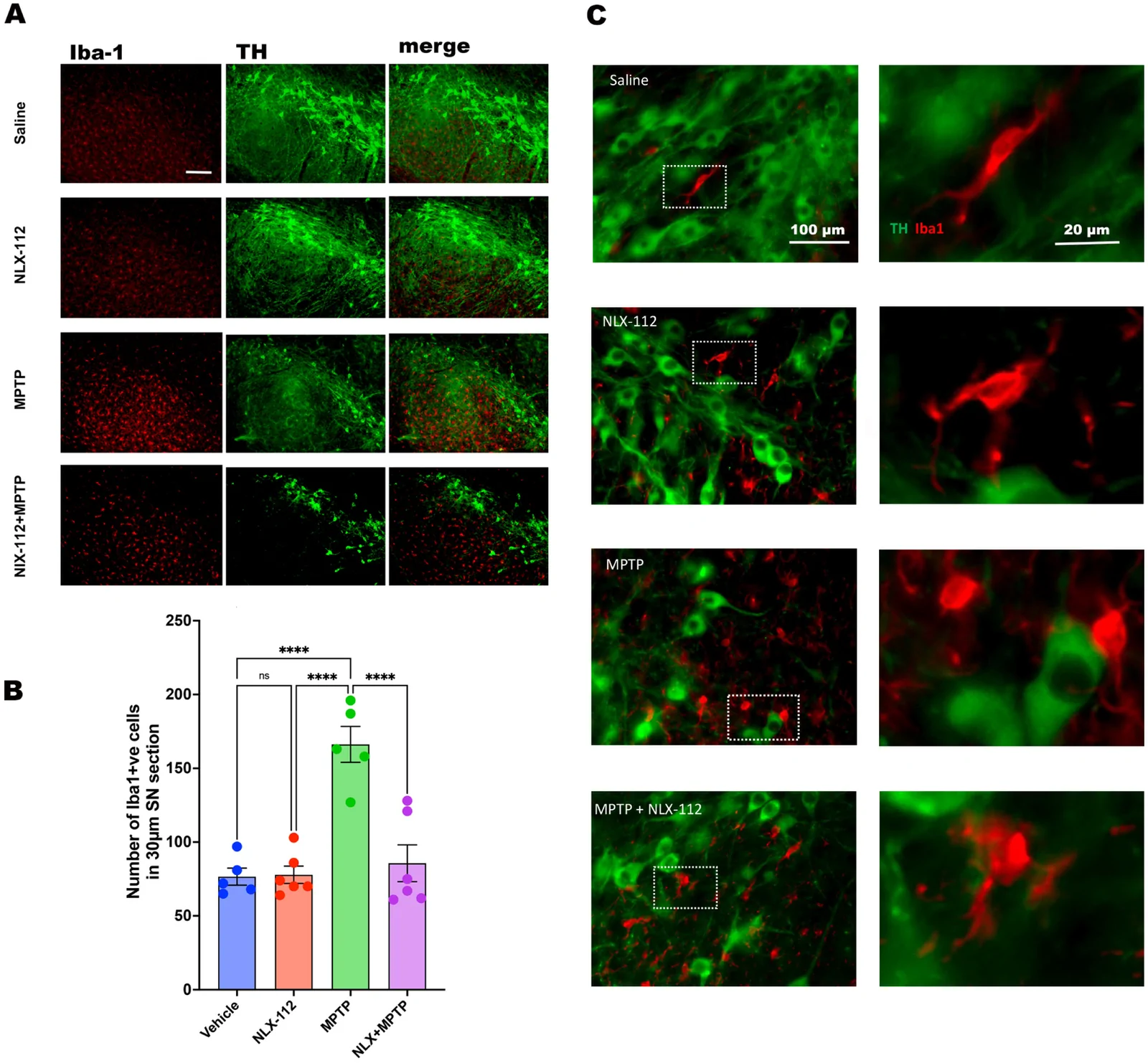
A) Reactive microgliosis in the SN stained with TH-ir (green) and ionized calcium-binding adapter molecule 1 (Iba1) (red) at 10× magnification. The white scale bar in the upper left photo represents 100 μm. **B)** Quantification of Iba1-ir cells in the SN. ***P < 0.0001, Tukey’s post hoc test following significant one-way ANOVA. *N* = 5–6 per group, bars are means; error bars are SEMs. In panel **C**, merged TH-ir and Iba-1 images for different treatments are shown in high magnification (40×).

### Reactive Astrocytosis and GDNF-GFAP Colocalization

In the SNc of mice treated with saline or NLX-112, or following MPTP treatment, there was a modest presence of GFAP-ir fibrillar cells (Figure 5A). While MPTP did not increase the number of these cells, the morphology of the cells displayed an “activated” state, developing prominent fibrillar end-feet. Double labeling with glial-derived neurotrophic factor (GDNF) showed occasional colocalization of GDNF-ir with GFAP-ir. In MPTP mice that were treated with NLX-112, cells immunoreactive for GDNF were more frequently associated with GFAP-ir, appeared to have a distinct astroglial morphology, and were significantly more frequently observed [F­(3, 35)=9.164, *P* < 0.0001, Figure 5B]

**Figure 5:**
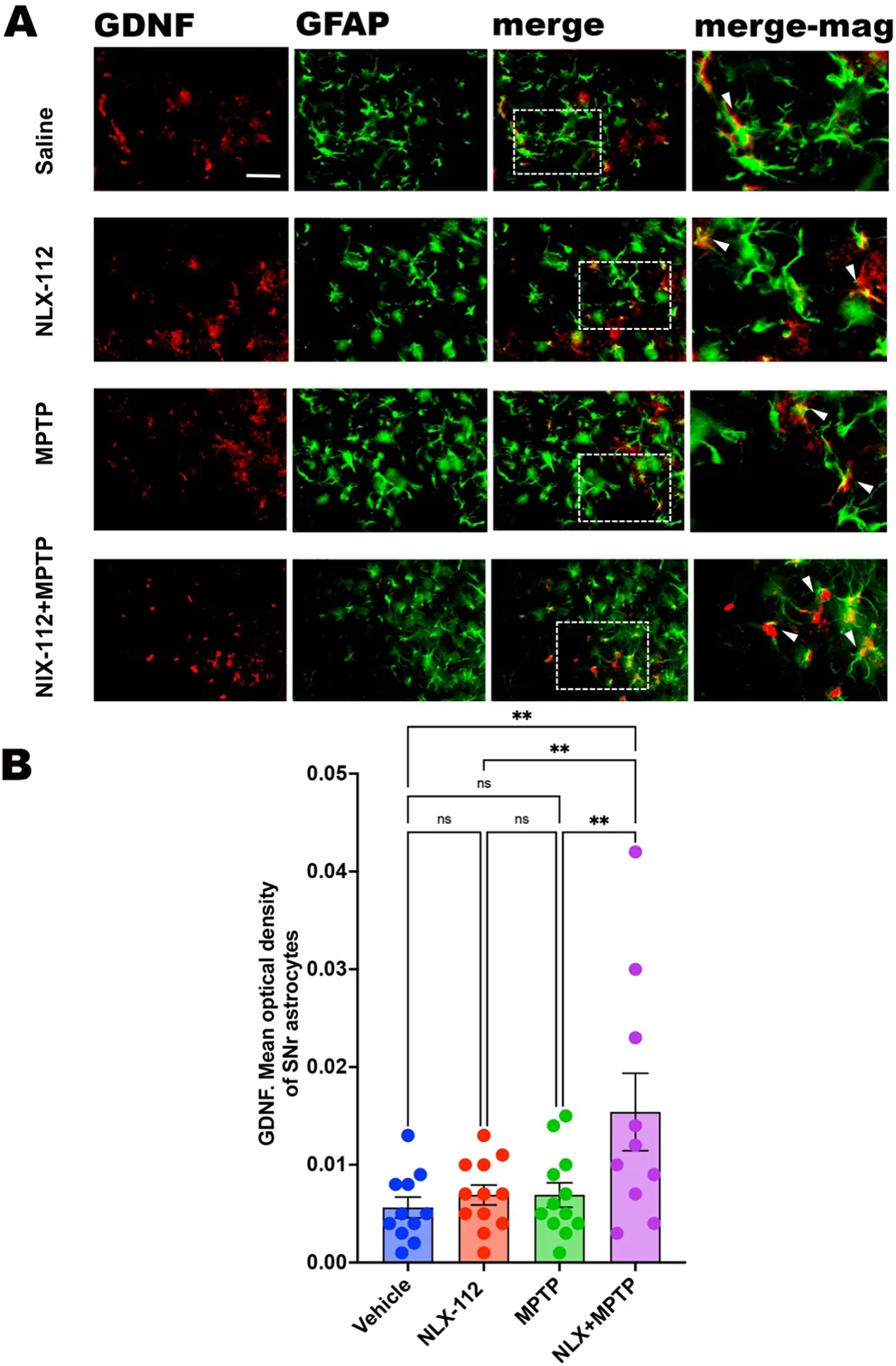
A) GFAP and GDNF colocalization in the SN at 40x magnification. Merge-mag: magnified portion of the merged image (scale bar, 50 μm). **B)** Quantification of colocalization of GDNF-GFAP in the SNr. Bars are means; error bars are SEMs. *N* = 10–12. *P < 0.05, **P < 0.001, ***P < 0.0001, Tukey’s post hoc test following one-way ANOVA, *n* = 10–12. The white scale bar in the upper left photo represents 50 μm.

In the striatum, very few GFAP-ir fibrillar astrocytic cells were observed in saline- or NLX-112-treated normal mice. There were few instances of GDNF-ir and GFAP-ir colocalization in vehicle (saline)-treated mice or following NLX-112 administration alone (Figure 6A). MPTP treatment markedly increased the number of GFAP-ir fibrillar astrocytic cells. In the MPTP-treated animals that received NLX-112, the number of GFAP-ir cells was significantly decreased [F­(3, 35) = 11.09, *P* < 0.0001; Figure 6B]. The number of GDNF-ir immunoreactive cells increased substantially following MPTP and further increased following MPTP + NLX-112 [F­(3, 35)=13.44, *P* < 0.0001, Figure 6C]. Colocalization of GFAP-GDNF in the striatum was increased by 110% following MPTP and was increased even further by 333% in the NLX-112 + MPTP group.

**Figure 6:**
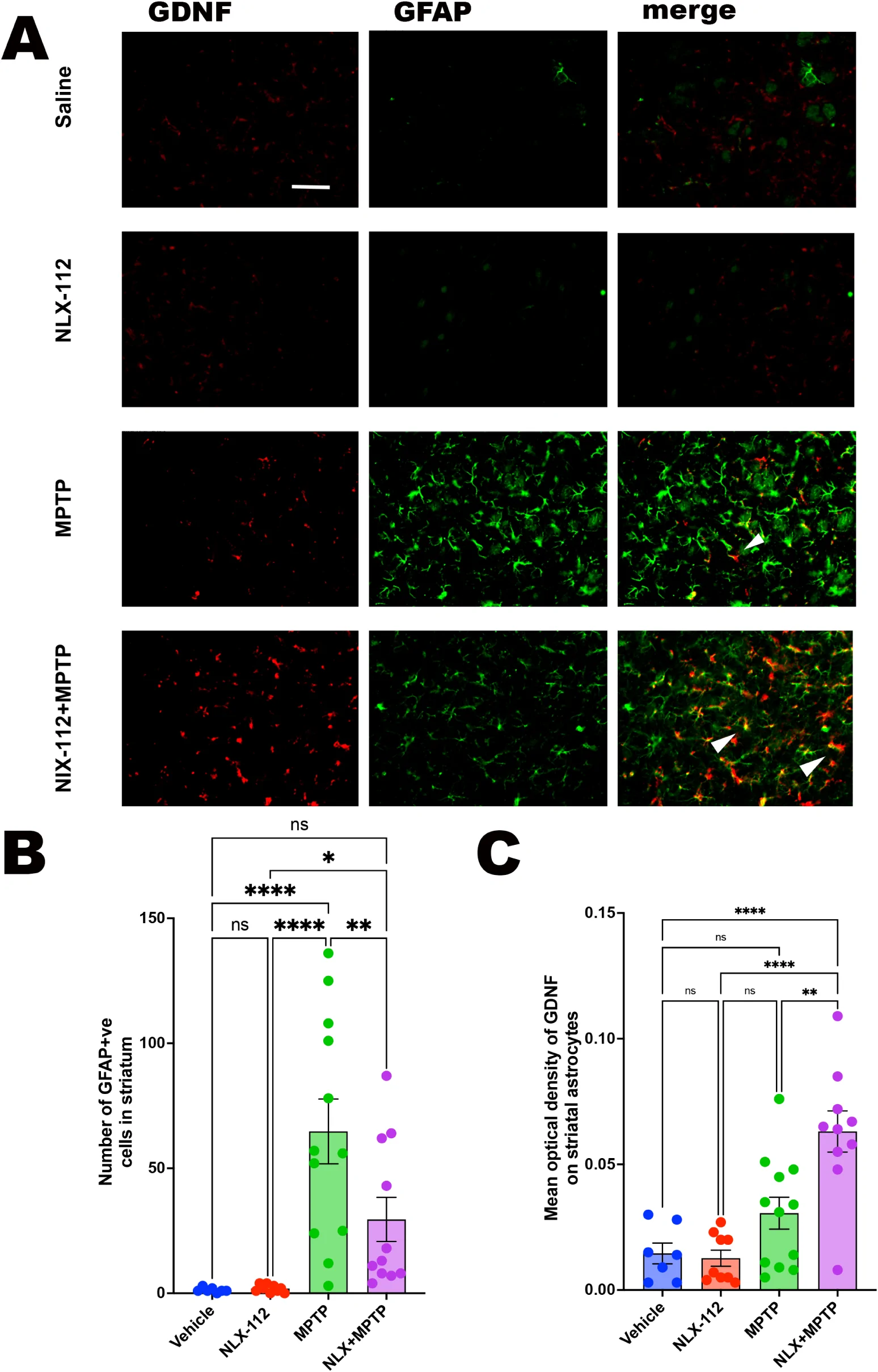
A) Representative images of GDNF and GFAP colocalization in the mid Caudate Putamen of saline, MPTP, NLX-112, and NLX-112+MPTP treated mice, 20× magnification. **B)** Quantification of GFAP immunoreactivity in the ventral striatum. **C)** Quantification of GDNF-GFAP colocalization on striatal astrocytes. *P < 0.05, **P < 0.001, ***P < 0.0001, ****P < 0.00001, Tukey’s post hoc test following one-way ANOVA. Bars are means; error bars are SEMs. *N* = 7–12 per group. The white scale bar in the upper left photo represents 50 μm. The white arrows indicate instances of colocalization.

## Discussion

Parkinson’s disease (PD) is a progressive neurodegenerative disorder characterized by the loss of dopaminergic neurons in the substantia nigra (SN), leading to motor dysfunction, cognitive decline, and nonmotor symptoms. To date, there is no approved therapy capable of halting dopaminergic cell loss or fully restoring neuronal function. In this context, the present study reveals that NLX-112, a selective serotonin 5-HT_1A_ receptor agonist, exerts neuroprotective activity via a mechanism which is nondopaminergic and mediated via glial cell line-derived neurotrophic factor (GDNF), a critical neurotrophic factor for the survival, growth, and differentiation of dopaminergic neurons. Of note, previous preclinical studies have shown promising neuroprotective and regenerative effects of GDNF, but clinical trials have produced inconsistent results.^28,29^ Thus, intermittent low-dose GDNF infusion increased striatal dopamine activity, as measured by PET imaging, but failed to confer significant improvements in clinical motor function over a 40-week period.^29^ Continuous infusion strategies also failed to demonstrate clear symptomatic benefits compared to placebo controls.^28^ A more recent approach combining convection-enhanced delivery (CED)—which improves the distribution of therapeutics throughout the putamen—with AAV2-GDNF gene therapy, a viral-based system designed to induce long-term local expression of GDNF^30,31^ demonstrated that this strategy was safe, well-tolerated, and maintained stable motor scores over a 45-month follow-up period in early trials involving 13 PD patients.^30,32^ These findings indicate that enhancing GDNF activity is a promising therapeutic approach but highlight the challenge of delivering adequate GDNF concentrations to target regions in the human brain while maintaining safety and tolerability.

### NLX-112 as a Neuroprotective Agent

The present study evaluates the selective 5-HT_1A_ receptor agonist NLX-112 in an MPTP mouse model of PD. Key outcomes observed include:

Sustained prolocomotor behavioral effects, indicating preservation of motor circuitry.
Reduction of microgliosis reflects attenuated MPTP-induced neuroinflammation.
Upregulation of endogenous GDNF from astrocytes supports dopaminergic neuron survival.
Partial preservation of the nigrostriatal dopaminergic pathway suggests structural neuroprotection.

These results reinforce preclinical and clinical findings highlighting GDNF as a potent neuroprotective factor in PD^33–38^ and point to selective targeting of 5-HT_1A_ receptors as a novel strategy to achieve GDNF-mediated neuroprotection.

A notable differentiation of NLX-112, compared with previous neuroprotective interventions, is that it targets endogenous GDNF upregulation rather than exogenous delivery, offering a potentially more physiologically relevant and sustained neuroprotective effect.

### Astrocytic GDNF Upregulation via 5-HT_1A_ Activation

Astrocytes are multifunctional glial cells that not only support metabolic and structural neuronal homeostasis but are also key sources of endogenous GDNF. The present study demonstrates that NLX-112 stimulates GDNF expression in GFAP-positive astrocytes, suggesting that astrocytic activation mediates much of its neuroprotective effects. Prior studies show that astrocytic SARM1 deletion in models of multiple sclerosis enhances GDNF-mediated neuroprotection,^39^ while ischemic neuronal injury elicits astrocytic GDNF release, promoting neuronal survival.^40^

It is established that astrocytes express 5-HT_1A_ receptors, which are typically dormant under basal conditions but become functionally active following neuronal injury or toxic insult.^15,41–45^ Activation of 5-HT_1A_ receptors triggers antioxidant release (e.g., metallothionein and S-100) and GDNF upregulation. NLX-112 alone did not increase GDNF levels in the absence of MPTP-induced injury, indicating that GDNF induction by NLX-112 is context-dependent. MPTP treatment activates astrocytes, as reflected by increased numbers of GFAP-immunoreactive cells, providing a cellular substrate for the upregulation of GDNF. Therefore, the observed NLX-112-mediated increase in GDNF likely requires astrocytic priming by neurotoxic or inflammatory signals, consistent with a mechanism in which the magnitude of the GDNF upregulation is proportional to astrocyte activation.

In the present study, immunohistochemical analyses revealed that GDNF secretion occurred only when NLX-112 treatment coincided with MPTP-induced neuronal stress, highlighting the necessity of a neurotoxic trigger for functional 5-HT_1A_ receptor engagement.

### Mechanisms of NLX-112 Neuroprotection

MPTP is converted by astrocytic MAO-B into MPP+, which enters dopaminergic neurons via the dopamine transporter (DAT). MPP+ inhibits mitochondrial complex I, leading to ATP depletion and reactive oxygen species (ROS) accumulation, and ultimately triggering apoptosis. In the case of NLX-112, we hypothesize that it engages astrocytic 5-HT_1A_ receptors to induce the secretion of GDNF, which then binds to RET receptors on dopaminergic neurons. RET activation restores mitochondrial function, enhances ATP production, mitigates ROS, and suppresses apoptotic pathways, thereby protecting neuronal integrity.^46^

Additionally, NLX-112 reduces microgliosis and neuroinflammation, creating a supportive microenvironment for neuronal survival. Together, these mechanisms preserve the structural and functional integrity of the nigrostriatal pathway.

### Behavioral Correlates of Neuroprotection

NLX112 administration improved locomotor activity and produced anxiolytic-like effects in MPTP-treated mice, as evidenced by increased exploration of the center of the open-field arena rather than the thigmotactic behavior typically associated with anxiety-like responses [22]. These behavioral effects persisted for at least 2 weeks after treatment cessation, indicating a sustained functional impact of 5-HT1A receptor activation.Behavioral improvements most likely result from selective engagement of cortical and striatal 5-HT_1A_ receptors, regions critical for motor control and mood regulation.^20,47^ Enhanced 5-HT_1A_ receptor sensitivity in MPTP-exposed mice likely amplifies the therapeutic response, as supported by PET imaging using ^18^F-NLX-112 and ^18^F-FDG studies, which revealed improved receptor functionality and brain energy metabolism following treatment.^48,49^

## Limitations of the Study

Some limitations should be mentioned: only male mice were used, and a comparison with female subjects would be warranted in future studies. A further limitation of the study is that the loss of the nigrostriatal dopaminergic pathway was assessed using TH-ir only. Although reductions in TH-ir can reflect dopaminergic neuron loss, the TH-ir phenotype is potentially reversible, raising the possibility that some neurons may have survived but downregulated their TH expression following MPTP exposure. However, earlier work using Fluoro-Gold prelabeled dopaminergic neurons demonstrated that true cell loss occurs following direct neurotoxin administration and that this degeneration can be prevented by a neuroprotective agent.^50^ Nevertheless, it remains possible that, in the present study, a proportion of the observed TH-ir reduction reflects phenotypic downregulation rather than complete neuronal loss.

Additional behavioral tests (i.e., balance beam, pole climbing, rotarod) could be carried out to further assess motor functions. While the MPTP model is a robust toxin-induced model of PD, additional studies are warranted using other models, such as alpha-synuclein-based ones. Finally, the dose of NLX-112 was chosen based on extensive previous studies of the compound, but it would be instructive to investigate the effects of additional doses, which may produce even more complete protection. Furthermore, while increased GDNF expression was demonstrated using immunohistochemical approaches, complementary quantitative measurements (e.g., ELISA or Western blotting) and direct mechanistic validation using GDNF-blocking strategies were not performed. Similarly, downstream analyses of mitochondrial function and apoptosis-related pathways were beyond the scope of the current work. In addition, quantitative assessment of astrocytic activation was performed in the striatum but not in the substantia nigra because of anatomical and technical limitations associated with robust quantification in this region. Future studies will be specifically designed to address these mechanistic questions.

## Conclusion

This study demonstrates that NLX-112 exhibits potent neuroprotective properties in an MPTP mouse model of PD. Protection is mediated through the attenuation of astrocytic and microglial activation, the upregulation of endogenous GDNF, and the preservation of nigrostriatal dopaminergic neurons. These cellular effects translate into sustained prolocomotor and anxiolytic-like behavioral benefits, suggesting that selective 5-HT_1A_ receptor agonists may represent a novel and promising therapeutic strategy for neuroprotection and disease modification in PD.

## Data Availability

All data generated and analyzed is available unrestricted and can be provided upon request.

## Abbreviations

5-HT: 5-hydroxytrptamine
^**18**^ **F-FDG**: fluorodeoxyglucose F^18^
CED: convection-enhanced delivery
ELISA: enzyme-linked immunosorbent assay
GDNF: glial cell line-derived neurotrophic factor
GFAP: glial fibrillary acidic protein
IBA-1: ionized calcium-binding adapter molecule 1
ir: immunoreactive
MPTP: 1-methyl-4-phenyl-1,2,3,6-tetrahydropyridine
PD: Parkinson’s disease
TH: tyrosine hydroxylase
PET: positron emission tomography
SNc: substantia nigra pars compacta
